# Outcome based subgroup analysis: a neglected concern

**DOI:** 10.1186/1745-6215-10-33

**Published:** 2009-05-20

**Authors:** Karim F Hirji, Morten W Fagerland

**Affiliations:** 1 Department of Epidemiology and Biostatistics, Muhimbili University of Health and Allied Sciences, Dar es Salaam, Tanzania; 2 Ullevål Department of Research Administration, Oslo University Hospital, Norway

## Abstract

**Background:**

A subgroup of clinical trial subjects identified by baseline characteristics is a proper subgroup while a subgroup determined by post randomization events or measures is an improper subgroup. Both types of subgroups are often analyzed in clinical trial papers. Yet, the extensive scrutiny of subgroup analyses has almost exclusively attended to the former. The analysis of improper subgroups thereby not only flourishes in numerous disguised ways but also does so without a corresponding awareness of its pitfalls. Comparisons of the grade of angina in a heart disease trial, for example, usually include only the survivors. This paper highlights some of the distinct ways in which outcome based subgroup analysis occurs, describes the hazards associated with it, and proposes a simple alternative approach to counter its analytic bias.

**Results:**

Data from six published trials show that outcome based subgroup analysis, like proper subgroup analysis, may be performed in a post-hoc fashion, overdone, selectively reported, and over interpreted. Six hypothetical trial scenarios illustrate the forms of hidden bias related to it. That bias can, however, be addressed by assigning clinically appropriate scores to the usually excluded subjects and performing an analysis that includes all the randomized subjects.

**Conclusion:**

A greater level of awareness about the practice and pitfalls of outcome based subgroup analysis is needed. When required, such an analysis should maintain the integrity of randomization. This issue needs greater practical and methodologic attention than has been accorded to it thus far.

## Background

Clinical trial data are frequently analyzed to identify subgroups of patients for whom a treatment is especially indicated. Known as subgroup analysis (SGA), it is, in theory, a useful venture. But the manner in which it has been done and reported in practice has generated widespread confusion and misleading claims.

Clinical trials, in the first place, rarely plan, or have an adequate sample size for SGA. Often done post-hoc, it is also regularly overdone, selectively reported, not interpreted with due caution, and performed without a relevant test for interaction. With a marked potential to yield unreliable assessments of the existence, direction and extent of treatment differences for a specific category of patients, the clinical message emanating from SGA is generally poorly justified, and may also foster unneeded research [1-13].

Inappropriate SGA, nonetheless, continue to surface. Warnings and contentions on the practice, consequently, persist in the medical journals. The exchange between Julian [14] and Sleight [15], and between Altman [16] and Feinstein [17], for example, typify the contrasting views on the topic.

In their seminal paper on this issue, Yusuf et al. [13] describe two types of subgroups. A subgroup delineated by baseline characteristics is called a proper subgroup while one demarcated by a post-randomization event or measure is an improper subgroup. Besides giving an indepth critical evaluation of the analysis of proper subgroups, they also issued a strong admonition against analyzing improper subgroups. To quote:

Analysis of improper subgroups, though seductive, can be extremely misleading, because a particular treatment effect may influence classification to the subgroup. Thus, an apparent subgroup effect may not be a true effect of treatment but rather the result of inherent characteristics of patients that led to a particular response or to the development of side effects.

In the eighteen years since this warning was issued, critical scrutiny of improper subgroup analysis has all but ceased. The extensive methodologic and clinical debate on SGA to date has essentially dealt with proper subgroups. At the most, only a brief warning on analyzing improper subgroups is given once in a while (for instance, Cook at al. [18]). In practice, on the other hand, it flourishes. The current literature is replete with such analyses done under a broad variety of guises, and their findings often impact therapeutic conclusions. An examination of the forms, frequency, validity and reliability of this practice is thus warranted.

This paper examines one category of improper subgroups, namely, those formed in relation to a specific outcome in a trial. Our focus is on the subsequent analysis of that subgroup with respect to another outcome. We define such an analysis as an outcome based subgroup analysis (OBSGA). In a cancer trial, for example, a quality of life measure is compared among the survivors. Or, in a trial of acute otitis media, the presence of antibiotic resistance is compared only among those in whom bacteria are isolated.

The specific aims of this paper are (i) to draw attention to the practice of outcome based subgroup analysis; (ii) to present a variety of pertinent examples from the literature; (iii) to show that the known pitfalls of baseline based SGA also apply here; (iv) to illustrate the specific analytic challenges posed by OBSGA; and (v) to propose simple alternative, unbiased methods of analyzing outcome based data.

## Results and discussion

First, we define two key terms. In OBSGA, the outcome determining the subgroup membership is called the index outcome, and an outcome on the basis of which treatments are then compared within that subgroup is a subsidiary outcome. The index outcome generally is the primary outcome in the trial. At times, however, it is not.

Next, we give six examples of OBSGA from the medical literature to illustrate the variety of ways in which it occurs.

### Example 1. Lung cancer chemotherapy

Sundstrøm et al. [19] randomized small-cell lung cancer patients to etoposide and cisplatin (EC, *n *= 218) versus cyclophosphamide, epirubicin, and vincristine (CEV, *n *= 218). The respective two- and five-year overall survival rates for EC were 14% and 5%, and for CEV, they were 6% and 2%, (log-rank test *p *< 0.01).

A quality of life (QOL) study based on several functional and symptom scales applied at nine time points (the subsidiary outcomes) was also done. Only 316 (72%) of the patients consented for it. Further, due to death and other reasons, the number of available cases dropped to 115 by week 54. At week 54, the QOL study included 13% (*n *= 29) of CEV, and 19% of EC (*n *= 42) randomized cases.

### Example 2. Cell transfusion in critical care

Does epoetin alfa reduce the need for red-cell transfusions in critically ill patients? Corwin et al. [20] examined this issue by randomizing 1460 cases in an intensive care facility to either epoetin alfa (*n *= 733) or placebo (*n *= 727). At 29 days, 46% of the former, and 48% of the latter had had a red-cell transfusion (Cochran-Mantel-Haenszel test *p *= 0.34).

Here, the number of red-cell units transfused was a subsidiary outcome with mean and median values only given for the transfused cases. Treatment-wise comparison for this outcome in this subgroup yielded a Wilcoxon-Mann-Whitney test *p *= 0.69.

### Example 3. Treatment strategies for stroke

Bernhardt et al. [21] is a phase II randomized trial of the safety and feasibility of very early mobilization with standard care (*n *= 38) as compared to only standard care (*n *= 33) for stroke patients. By month 3, 21% (8) of the former and 9% (3) of the latter had died (Fisher's exact test *p *= 0.20).

The numbers of serious and nonserious adverse events among the three month survivors were used as subsidiary outcomes. The Poisson regression *p*-value for the former was 0.85 and for the latter it equaled *p *= 0.04.

### Example 4. Probiotics and atopic dermatitis

Does Lactobacillus GG (LGG) prevent the development of atopic dermatitis (AD) in newborns? Kopp et al. [22] randomized 105 pregnant women from families with an atopic disease to either LGG (*n *= 54) or placebo (*n *= 51) to address this issue. Occurrence of AD by the age of two was the primary (index) measure. Of the 50 LGG and 44 placebo children completing the trial, 28% and 27%, respectively, were diagnosed with AD (chisquare test *p *= 0.93).

The severity of AD after two years was the subsidiary outcome. Median values on the Scoring Atopic Dermatitis Index (LGG = 18.5 & placebo = 22.5) were reported and compared only for the children diagnosed with AD (Wilcoxon test *p *= 0.80).

### Example 5. Antibiotics for acute otitis media

McCormick et al. [23] randomized children with nonsevere acute otitis media to either watchful waiting (WW, *n *= 111) or immediate antibiotics (IABX, *n *= 112). Carriage of resistant bacterial strains at day 12, one of the four primary outcomes, occurred in 16% (18) of the IABX group and 39% (43) of the WW group (our chisquare *p *< 0.01).

With culture growth status as the index outcome, two subsidiary outcomes based on it were: (i) the count of antibiotics to which the bacterium was resistant, and (ii) the level of resistance to penicillin. With the data for (i) shown in Table 1, among the culture positive cases, the WW group had a lower proportion of cases resistant to a larger number of antibiotics (chisquare *p *< 0.02). An analysis of resistance to penicillin yielded a similar finding.

**Table 1:** Number of non-sensitive antibiotics

Number	IABX	WW
0	0	13
1–3	8	18
4–6	10	12

Negative Culture*	94	68

Source: Table Six. McCormick et. al. [23]

* Includes 5 cases with missing data.

### Example 6. An arrhythmia suppression trial

Cowley et al. [24] randomized 48 suspected acute myocardial infarct patients to lorcainide, an antiarrhythmic drug, and 47 to a matching placebo for six weeks. The index, though not the primary, outcome was survival to week 6. There were sixteen subsidiary outcomes derived from 24-hour ECG records at three time points: first 24 hours, day 6, and during week 6. Occurrence of severe arrhythmias (SA) was the main subsidiary outcome as well as the primary outcome of the trial. The main time point for this was, however, not uniquely or clearly specified.

Due to death or loss from the study, 80 of the 95 patients were available at the first time point; 78 at the second, and 58 at the third. By week 6, 19% (9) lorcainide and 2% (1) placebo patients had died (No statistical significance is ascribed; our *p *= 0.01).

In the 48 subsidiary comparisons, five were significant at the 0.01 level. At week 6 in particular, 8% (2) of the available 24 lorcainide patients, and 38% (13) of the 34 available placebo patients had SA. (The former is erroneously stated as 13%, and no *p*-value is given; our *p *= 0.01).

Looking at all their data, the authors conclude:

Lorcainide was shown to be an effective anti-arrhythmic agent. The study was not designed to evaluate the effect of lorcainide on survival, ...

Table 2 summarizes these six examples. In each case, the analysis of a subsidiary outcome was done within a subgroup determined by a specific level of the index outcome. They constitute but a small facet of the wide variety of outcome based subgroup analyses that continue to be reported in the clinical trials literature.

**Table 2:** Varieties of index and subsidiary outcomes

Example	Disease	Index Outcome	Subsidiary Outcome
1. Sundstrøm et al. [19]	lung cancer	survival	quality of life
2. Corwin et al. [20]	critical illness	red-cell transfusion	number of units transfused
3. Bernhardt et al. [21]	stroke	survival	adverse events
4. Kopp et al. [22]	AD*	occurrence of AD	severity of AD
5. McCormick et al. [23]	AOM^†^	isolation of bacteria	level of antibiotic resistance
6. Cowley et al. [24]	AMI^‡^	survival	severity of arrhythmia

* AD: atopic dermatitis; ^† ^AOM: acute otitis media; ^‡ ^AMI: acute myocardial infarction

### Practical Concerns

A crucial distinction between baseline and outcome based subgroups is that while the size and composition of the former are determinable when randomization ends, those of the latter are not until the end of the trial. The latter also does not allow a separate subsidiary outcome analysis for all the subgroups. A QOL comparison among the dead, for example, cannot be done. These differences notwithstanding, the typical outcome based subgroup analyses seen in the literature tend to exhibit most of the problems associated with baseline based SGA that we listed in the introduction.

#### Reduced Power

OBSGA is done for a subset, at times quite a small subset, of the trial sample. In Example 1, OBSGA at 54 weeks was done for less than 20% of the cases; in Example 2, for less than 50%, and in Examples 4 and 5, for less than 30%. In Example 6, about 60% of the sample was available for the evaluation at the final time point. The power to detect clinically meaningful effects, especially when the study has a moderate or small sample size to start with, is then reduced considerably.

#### Overdone Post-Hoc Analysis

None of the Examples 1 to 5 clearly states if the OBSGA was pre-planned or allowed for in the sample size calculation. In Example 6, the subsidiary outcome was the primary outcome, with the level of statistical significance apparently at 0.01. But the outcome was measured in a number of ways, a large volume of data collected at several time points, and the rationale for not designating survival also as a primary outcome is subject to question. The large number of subsidiary analyses done in such cases raises the false positive error rate, and at times, does so substantially. In Example 1, QOL was evaluated at nine points in time, using 14 scales. A total of 119 significance tests appear in Tables One-Five of the paper. A similar scenario prevails in Examples 3 and 6. Such analyses resemble fishing expeditions for statistical significance.

#### Selective Reporting

With multiple, unplanned, and data driven analyses, the potential for selective highlighting of only the significant findings is enhanced. Examples 3 and 6 appear to exhibit this tendency. Without access to trial protocol and records such practices cannot be verified.

#### Over Interpretation

Reduced power, biased comparison (see below), and a high false positive rate render the conclusions of an OBSGA as tentative, at best. Yet, authors do not interpret them with caution. In all these six examples, such findings attain a greater credence than they deserve.

### Biased Analysis

The key issue for SGA is the presence, size and direction of an interactive effect between treatment and a risk factor. That effect may occur in a masked fashion, and is not easy to detect unless the sample size is quite large. For baseline determined subgroups, we may analyze the trial data for interaction even when close baseline balance prevails. But this needs to be done with a preplanned appropriate test that includes all the randomized subjects. Otherwise, the exercise can do more harm than good.

For outcome based subgroups, the situation is more complex. In the first place, the baseline factors exercising main and interactive effects on treatment may be different for the index and subsidiary outcomes. Furthermore, these two outcomes may also impact each other. For example, poor QOL may, independent of other factors, lower the chance of survival. In such a situation, despite randomization, patients on distinct treatment arms within an outcome based subgroup are not likely, even on average, to be similar in terms of baseline features. Furthermore, biases introduced during the course of the trial like differential care, follow up and evaluation can influence the composition of treatment subsets within the subgroup as well.

To illustrate how such interactive effects are manifested, we present, in Table 3, data from six hypothetical trials (labeled Trial A to Trial F) of an antiarrhythmia drug. The arrhythmia suppression trial of Example 6 forms the backdrop for these data. Each trial randomized 200 patients to either an antiarrhythmic drug (*n *= 100) or placebo (*n *= 100). Two outcomes were recorded six weeks later: survival and the level of arrhythmia (SA or Not SA).

**Table 3:** Baseline status and week 6 outcome

	Baseline: No SA		Baseline: SA			Baseline: No SA		Baseline: SA	
Week 6 Status	Drug	Placebo	Drug	Placebo		Drug	Placebo	Drug	Placebo

	Trial A					Trial D			
Alive & No SA	40	40	0	0		10	20	10	0
Alive & SA	10	10	0	25		30	40	30	0
Dead	0	0	50	25		10	4	10	36

	Trial B					Trial E			
Alive & No SA	25	25	15	15		0	20	20	0
Alive & SA	0	25	10	10		40	4	20	36
Dead	25	0	25	25		10	40	10	0

	Trial C					Trial F			
Alive & No SA	35	30	5	10		10	20	10	0
Alive & SA	5	20	5	15		40	34	20	6
Dead	10	0	40	25		0	10	20	30

Note: Trials A, B, C ⇒ exact baseline balance; Trials D, E, F ⇒ moderate baseline imbalance

We start with Trials A, B & C. In each of them, 50 drug and 25 placebo cases had died by week 6 (*p *< 0.01). Further, 10 of the 50 drug survivors, and 35 of the 75 placebo survivors had SA (*p *< 0.01). In line with Cowley et al. [24], we may then infer that the drug, though without a survival benefit, is an effective antiarrhythmic.

Now suppose the baseline arrhythmia levels in Trials A, B & C were as shown in Table 3. In each, there is exact baseline balance in terms of this factor, with the baseline odds ratio for SA (BOR(SA)) between treatment and control groups equal to 1.00. Despite this and identical overall outcomes, the outcome patterns within the subgroups defined by baseline severity of arrhythmias were quite different in the three trials.

In Trial A, for those without initial SA, the drug and placebo had identical effects on survival and progression to SA at week 6. For those with initial SA, however, the drug reduced the week 6 survival rate from 50% to 0%. The excess of SA in the placebo group at week 6 does not reflect a beneficial effect of the drug but simply the number of patients with initial SA who were still alive. For the survivors, the BOR(SA) is 0.00, indicating a potential selective interactive effect at work.

In Trial B, on the other hand, the drug seems to be harmful only for those who did not have SA at the start. While 50% of placebo cases without SA at the outset tend at the worst to develop SA by week 6, 50% of similar cases on the drug die by week 6. Possibly for those who first develop SA while on the drug, the arrhythmias tend to be of sufficient severity as to cause death. The drug thus harms patients without SA, but otherwise is not different from placebo. The BOR(SA) for the survivors is 2.00, indicating the possible presence of a different type of selective interactive effect.

In Trial C, the drug appears to affect a higher death rate than placebo whether or not the subject had SA at the start. Similarly, among the survivors, those on the drug had a lower proportion of SA compared to placebo irrespective of baseline status, though the magnitudes of these difference are not the same. And, among the survivors in this trial, the changed BOR(SA) of 0.50 may denote an interactive effect.

Now consider Trials D, E & F. The overall results for these three trials are also identical. In each, the death rate at week six on the drug was 20% while that on placebo was 40% (*p *< 0.01); among the survivors, 60 out of the 80 (75%) in the drug arm had SA at week 6 while 40 out of 60 (66%) in the placebo arm had this condition (*p *= 0.28). The usual analysis thereby indicates that the drug improves survival but does not noticeably improve arrhythmia severity among the survivors. However, in each trial, a moderate but statistically significant baseline difference prevailed: 50% of the drug arm had SA at baseline but only 36% of the placebo cases were so affected (*p *< 0.05). The complete sample BOR(SA) for each trial was 1.80. Hence, the directions of the observed differences as well as the higher proportion of the more severely afflicted cases in the drug group needs to be kept in mind when drawing our conclusions.

Trials D, E & F also exhibit distinct interactive scenarios (Table 3). In trial D, the death rate among those without baseline SA is higher for the drug group than for placebo (20% versus 6%) but the situation is reversed for those with SA at baseline (20% versus 100%). But in terms of SA among the survivors, the difference between the two groups is a marginal one for those without baseline SA and indeterminate for those with baseline SA. The pattern for the two outcomes viewed in a combined way for the drug also does not seem to depend on baseline status. Among the survivors, the BOR(SA) has risen to *8 *(note, it was 1.80 for all the randomized cases).

In Trial E, the results for survival are the opposite of those for Trial D. Here the death rate among those without baseline SA is lower for the drug group than for placebo (20% versus 62%). The antiarrhythmic effect of the drug is much more pronounced among those with baseline SA compared to those without baseline SA. The BOR(SA) for the survivors of Trial E is 0.67.

Trial F, on the other hand, differs from Trials D & E, in that the drug has a lower death rate compared to placebo for those with baseline SA and those without it. But the antiarrhythmic benefits of the drug seem more pronounced among those with baseline SA. The BOR(SA) for Trial F, at 5.4, is also higher than the 1.80 value for the overall sample.

The key message from these hypothetical trials is that the issue of interaction in outcome based subgroups is a more complex affair than for baseline subgroups. Whether baseline balance prevails or not, similar overall data for the index and subsidiary outcomes may arise from quite distinct and even conflicting underlying scenarios.

The approach in the overall analyses of the subsidiary outcome in these trials is the same as in all the real data examples we gave. In a QOL study, Trials A, B & C would thus imply that the drug may reduce survival but at least improves the QOL among the survivors. Our hypothetical examples show such an interpretation of subsidiary outcome data, in particular, needs to consider the possibility of a complex web of relationships linking baseline factors and treatment on the one hand to the index and subsidiary outcomes on the other, and those linking the two outcomes to each other as well. In part, this is indicated by the fact that in each of the six trials, the distribution of the baseline risk factor in treatment subsets in the outcome based subgroup is different, and often very different, from the pattern at randomization. Unlike baseline subgroups, this is not just a matter of chance but a possible systematic consequence of such interactive effects. The typical analysis of the subsidiary outcome thereby also carries a strong inherent potential for bias.

To conclude this section: The impact of key prognostic factors, and their linkages to the index and subsidiary outcomes are often unknown. Randomization does not mitigate outcome based imbalances. With a marked possibility of bias, the typical OBSGA seen in practice is unreliable. Whatever the extent of initial confounding, a separate second outcome analysis for a subset of subjects determined by the value of an index outcome is necessarily suspect [13].

### Alternative Analyses

Subsidiary outcomes like severity of arrhythmias, QOL and the others noted in our examples often are important clinical outcomes. Appropriate approaches for the analysis of such outcomes are hence needed.

It is now recommended that the analysis of baseline based SGA, when warranted by design, should be done with an appropriate test for interaction. That, however, does not resolve all the issues. First, these tests have low power. Second, the distinction between biologic and statistical interaction, and the choice of a metric to measure interaction are important matters that need further resolution.

The root of the concern with outcome based subgroup analysis is also a possible presence of complex interactive effects. However, its resolution does not seem to lie in a search for a test for interaction. First, the relevant baseline factors and the manner in which they impact the two outcomes are not often known. Second, the key question for outcome based analysis of the overall data is not so much how to directly assess the presence of such interactive effects but rather how to adjust, in an unbiased way, for the impact and results of the index outcome when analyzing the subsidiary outcome. That is, when considering the severity of arrhythmias or QOL among the survivors, should we adjust for the difference, if any, between the treatment groups in terms of death rates? If so, how should we do it?

At first sight, we may regard this as a missing data problem, and apply one of the several available imputation methods to deal with it [25]. The two texts on the analysis of QOL data, in particular, raise the issue of missingness through attrition, non-response or death [26,27]. But, they do not go on to describe how to address outcome (death) based missingness. Also note that even when missingness is due to drop out, loss to follow up, or similar reasons, most published QOL analyses employ complete case analysis, and not imputation, to deal with the problem [28].

The new step would thus be to impute the values of the subsidiary outcome for cases in whom the index outcome generally precludes their existence. A major advantage would be the inclusion of all randomized subjects in the analysis and removal of baseline factors related bias. There are, however, two disadvantages here. One, the usual missing data methods are predicated on assumptions like missing at random that cannot be justified in the present context. According to Yusuf et al. [13], and as illustrated earlier, they are unlikely to hold. Two, for OBSGA, we have values that are not only unknown but also unknowable. To impute a low or moderate quality of life or arrhythmia level to a dead person does not appear appropriate. If you are dead, you are dead.

Another approach for OBSGA is to explicitly adjust outcome based comparisons in terms of potential confounding factors. In theory, this approach has less potential for bias than simply assuming that the unknowable values are missing at random. In practice, though, several hurdles remain. One, those factors are rarely known in advance. Attempts to identify them post hoc will enhance the false positive rate and turn the exercise into a fishing expedition. Two, outcome related subgroups are often small, and their sizes cannot be planned in advance. Three, for the purpose of adjustment, we need a statistical model that not only incorporates the interactive impact of the confounding factors on the index and subsidiary outcomes but also the relation between the two outcomes. Some conditional probability models that explicitly adjust for post-randomization variables have been developed, and may be adapted to this task [29]. However, this option requires more work to produce realistic and interpretable models, develop analytic strategies, and test them with real and simulated data.

Analyzing outcome based data thereby poses a distinctive challenge in terms of methods and interpretation. Comparing within outcome based subgroups can be unreliable even when baseline confounding appears absent. Under these circumstances, we suggest two simple strategies to avoid bias. Both of them take off from the observation that by excluding some of the randomized cases, OBSGA violates the intent-to-treat principle. To remedy this, we perform the subsidiary outcome analysis for the treatment groups as randomized. But unlike the classic missing data approach whereby subsidiary outcome values for the cases for whom they cannot be measured are ascribed by positing missingness at random or a similar mechanism, we ascribe these values on clinically or practically appropriate grounds.

Let us denote the usual separate analysis of an outcome based subgroup as Analysis I. Then, the two alternatives we suggest are as follows:

Analysis II: Ascribe the worst or best possible outcome, as clinically appropriate, for the subsidiary outcome to the subjects excluded by Analysis I. In a QOL assessment, for example, the dead cases are ascribed the lowest possible QOL score.

Analysis III: Ascribe a lower or higher level of outcome than the worst or best possible level, as clinically appropriate, of the subsidiary outcome to the subjects excluded by Analysis I. In a QOL assessment, the dead subjects get a QOL score lower than the lowest possible QOL score.

Analyses II and III thus resemble the best or worst outcome imputation used in the intent-to-treat analysis context. Their advantages are the inclusion of all subjects as randomized, control of the bias of Analysis I, and possibly higher power due to the larger sample size. Further, the findings of these analyses should be taken in conjunction with the analytic results for the index outcome.

Before turning to the specifics, we note a distinction. These approaches may be seen as constructing a composite outcome [30]. In a sense, this is true; for we do use the survival (index) outcome to extend the construction of the QOL (subsidiary) outcome. The crucial difference is that we do not seek a single outcome for the trial. We analyze these outcomes separately. The index outcome is, however, incorporated into the analysis of the subsidiary outcome.

The first practical question is how to choose between Analysis II and Analysis III. At times, the types of outcomes in the trial make that choice clear. Suppose the index outcome is the occurrence of an event, and the subsidiary outcome is the number of (the same) events among those for whom the event occurred. In that case, we prefer Analysis II with a zero value for those excluded by Analysis I. But at other times, the choice is not clear cut, and can affect the interpretation. In our view, that choice needs to made in consultation with subject matter experts. The second question pertains to the specific analytic technique to use. That, we hold, should reflect the nature of the data. Thus, for an ordered subsidiary outcome, a trend type of analysis is often better. The third question is the assignment of the lowest score for Analysis III. That matter also needs consultation with subject matter experts. And, furthermore, all these choices need to be made *a priori *and written into the data analysis protocol.

Now, we consider our examples. We set aside Examples 1 & 3 due to complexity of the analysis given in the respective papers and non-availability of the requisite data to perform our analyses. Examples 2 & 5 fall in the category where Analysis II is clearly the analysis of choice. These are reanalyzed first.

Example 2: The comparison of the number of red-cell units transfused done in the paper excluded subjects who were not transfused. Analysis II includes them with this number put at zero. This yields *p *= 0.45 with the *t*-test, a result similar to the one reported. In part, this is due to the fact that the treatment groups had similar index outcomes; had that not been the case, the two analyses could have differed markedly.

Example 5: Analysis II puts the count of resistant antibiotics for culture negative cases at zero. Analysis I uses the first three rows of Table 1, giving *p *< 0.01 for trend. Analysis II adds the last row to the first, producing a trend *p *= 0.14. Analysis II hence gives a more guarded conclusion compared to Analysis I, while avoiding its bias.

When the index and subsidiary outcomes differ in substance, like survival and QOL (Example 1), or survival and arrhythmia level (Example 6), choosing between Analysis II and Analysis III poses a particular challenge.

Example 4: Analysis II includes those who did not contract AD by putting their AD severity level at the lowest value, and Analysis III would chose a lower score. As the relevant data are not given, we cannot do this analysis.

Example 6: The drug significantly lowered the week 6 survival rate. Analysis I declares the rate of SA significantly smaller for the drug than for placebo (*p *= 0.01). For Analyses II and III under the worst-case intent-to-treat approach, the lost to follow up cases were combined with the dead cases in each group. Analysis II then contradicts Analysis I (*p *= 0.91), and so does Analysis III (*p *= 0.26). Analysis II possibly has a relatively unclear clinical message. The short follow up time may have clouded the results as patients with SA remained at higher risk for death. Analysis III, with the index (dead) cases a separate category, appears better.

Trials A, B & C: Analysis II combines the dead with alive & SA. In both groups, 60 out of 100 were dead or had SA, and 40 were alive without SA (*p *= 1.00). Analysis III based on a three level ordered outcome shows a marginally significant trend (*p *= 0.05). The drug induces higher fatality at six weeks, but among the survivors, a smaller proportion on it have SA. This is not too different from Analysis I, but the bias is avoided.

Trials D, E & F: The drug confers a significant survival benefit at week 6 (*p *< 0.01). For the rate of SA, Analysis I shows that the drug group is not that different from placebo (*p *= 0.28). Analysis II reiterates this message (*p *= 1.00), but Analysis III finds a significant trend (*p *= 0.04).

While avoiding the bias of Analysis I, Analyses II and III may produce a similar or a different message. Which one is more reliable? None, in our view, necessarily has higher power than the other. The nature of the specific subsidiary outcome and the statistical techniques used as well influence the answer. In Trial A, for example, pooling the dead with those who were alive but with SA gave a coarser outcome variable, and seems to have reduced power. But with different trial results that may not be so. Also, if, on the other hand, the subsidiary outcome was measured on a five ordered categories scale, Analysis II may be advisable. More studies are needed before we can lay out the conditions where Analysis II is preferrable over Analysis III, or vice versa.

The resolution of such questions requires simulation studies utilizing appropriate statistical models that incorporate the complex web of interactive effects possibly at work. Further, such studies need to compare these analyses with one based on a relevant multivariate conditional probability model for such data. Avenues for sensitivity analysis like the best or worst case scenarios to check the robustness of the alternatives we propose, and guidelines for assigning the subsidiary outcome scores also need to be developed.

Ultimately, the basic issues at hand are not just statistical issues but ones that also depend on patient values and preferences, and need input from clinicians in the field. Is death worse than a very poor quality of life, or very severe disease? Or is it more or less the same? How much relative weight does one put on death compared to life with extreme pain and suffering?

These are not easy questions. Yet, all analyses of outcome based data, implicitly or explicitly, posit and provide some form of an answer to them. Analysis I does so implictly and in a biased manner. Analyses II and III posit the issue more explicitly and provide an unbiased way to deal with it. However, more research and ethical consultations are required to produce field specific satisfactory answers to them.

## Conclusion

Analysis of a second outcome for cases at one level of an initial outcome is a form of improper subgroup analysis. In practice, it manifests most of the problems that have been noted with baseline based subgroup analysis. Also, as the subgroups are outcome determined, there is an intrinsic potential for bias associated with it. We have illustrated that conclusions based on it can be unreliable whether or not baseline confounding exists, and a particular conclusion may simultaneously reflect quite distinct underlying scenarios.

The guidelines in Wang et al. [12] for the usual SGA thereby need to be applied to outcome based subgroup analysis as well. When warranted, OBSGA has to be planned in advance, allowed for in sample size computation, use an adjusted *a*-level, and follow an appropriate method that accords with the intent-to-treat principle. Furthermore, due caution should always be exercised in interpreting its findings.

Our preliminary survey shows that OBSGA is frequently undertaken in the medical literature. Yet, it is not recognized as such. The strong warning issued against it by Yusuf et al. [13] has been ignored. In this paper, we once again draw attention to the practice and show some of the varied forms it assumes in clinical trial papers. Surveys done across different medical fields are, however, needed to document the extent and nature of the practice.

We have provided two simple alternatives that avoid bias, maintain the integrity of randomization and follow the intent-to-treat principle. Instead of excluding cases based on the index outcome, we incorporate them in the analysis of the subsidiary outcome in a clinically meaningful and statistically appropriate manner. In a cancer trial with survival and QOL as the outcomes, this implies that while the survival analysis is done in the usual manner, for comparing QOL, the dead cases are given the worst possible, or a lower QOL score.

These, however, are preliminary recommendations. Detailed studies with real and simulated data to assess their accuracy and precision in a variety of circumstances are needed. We hope our work will promote the needed research on the issue of outcome based subgroup analysis.

In conclusion: A cartoon shows a dentist boasting that his patients have 50% fewer cavities. His colleague then retorts, "That's because they have 50% fewer teeth" (Moore [31], page 161). Talking of the former without the latter can mislead; that is the key flaw of outcome based subgroup analysis. In principle, statisticians are well aware of this problem. Yet, a form of analysis with such a flaw has, despite the warning given by experts in a leading journal, entrenched itself in the clinical trial literature. It is time a further critical look at it was undertaken.

## Methods

This paper is not a comprehensive survey of OBSGA. Nor is it a technical work on statistical methodology. Rather, it has been written to highlight, illustrate, explain, reflect on and provide reasonable preliminary solutions for a crucial but neglected issue.

Our examples of OBSGA are derived from an ad hoc search of medical journals. From the cases found, we selected six that well illustrate the diverse manner in which it occurs. They are not presented as representative cases of OBSGA. We also use these examples to illuminate the similarities and differences between outcome and baseline based subgroup analyses. For each example, we noted the index and subsidiary outcomes, and summarized the analysis of the subsidiary outcome given in the source paper. Taking the one example where detailed data were available, we constructed six artificial data sets to argue how the typical way in which subsidiary outcomes are analyzed is not only biased but also may be invalid and perhaps derive from contrasting underlying situations. Some of the hypothetical scenarios are extreme, but they have been chosen to make the point clearly; other scenarios consistent with the overall results can be constructed as well.

Unless noted otherwise, the *p*-values in the Biased Analysis and the Alternative Analyses subsections are computed from the regular chisquare test. For trend analysis, the Cochran-Armitage trend test was used. All *p*-values computed or cited from the literature have been rounded to two decimal digits. For ease of exposition, we do not give confidence intervals.

For the hypothetical trials, the extent of baseline imbalance in terms of a risk factor was measured by the ratio of the odds of the high level of this factor between treatment and control groups at baseline. The disparity in this ratio between the survivors and the whole sample is used as an indicator of underlying interactive effects. The difference in the odds ratio is not necessarily the best measure of an interactive effect, and we also need to distinguish between biologic and statistical interaction. In the absence of a model linking the index and subsidiary outcomes to baseline factors and each other, our odds ratio comparisons are mainly meant to raise awareness of interactive effects, and not to provide a firm measure for them.

In the analysis we present, we employ a binary subsidiary outcome. However, our arguments readily extend to multilevel and continuous subsidiary outcomes.

## Competing interests

KFH and MWF have no competing interests in relation to this study.

## Authors' contributions

KFH conceived the study, produced an initial draft, and worked on the production of final draft. MWF searched for examples, checked the computations, and worked on the production of final draft. Both authors read and approved the final manuscript.
